# Healthy Patients Are Not the Best Controls for Microbiome-Based Clinical Studies: Example of Sjögren’s Syndrome in a Systematic Review

**DOI:** 10.3389/fimmu.2021.699011

**Published:** 2021-07-29

**Authors:** Elise Doaré, Geneviève Héry-Arnaud, Valérie Devauchelle-Pensec, Guillermo Carvajal Alegria

**Affiliations:** ^1^Rheumatology Department, Reference Centre of Rare Autoimmune Diseases, Cavale Blanche Hospital and Brest University, INSERM UMR 1227, Brest, France; ^2^UMR1078, Génétique, Génomique Fonctionnelle Et Biotechnologies, INSERM, Université de Brest, EFS, IBSAM, Brest, France; ^3^Centre Brestois d’Analyse du Microbiote, Hôpital La Cavale Blanche, CHRU de Brest, Brest, France

**Keywords:** oral microbiota, gut microbiota, Sjögren’s syndrome, sicca, dysbiosis

## Abstract

**Introduction:**

It has been hypothesized that gut and oral dysbiosis may contribute to the development of primary Sjögren’s syndrome (pSS). The aim of this systematic review was to assemble available data regarding the oral and gut microbiota in pSS and to compare them to data from healthy individuals and patients with dry symptoms without a diagnosis of Sjögren’s syndrome or lupus disease to identify dysbiosis and discuss the results.

**Methodology:**

Using the PRISMA guidelines, we systematically reviewed studies that compared the oral and gut microbiota of Sjögren’s patients and controls. The PubMed database and Google Scholar were searched.

**Results:**

Two-hundred and eighty-nine studies were found, and 18 studies were included: 13 referred to the oral microbiota, 4 referred to the gut microbiota, and 1 referred to both anatomical sites. The most frequent controls were healthy volunteers and patients with sicca symptoms. The most common analysis method used was 16S-targeted metagenomics. The results were mostly heterogeneous, and the results regarding diversity were not always in accordance. Dysbiosis in pSS was not confirmed, and reduced salivary secretion seems to explain more microbial changes than the underlying disease.

**Conclusion:**

These heterogeneous results might be explained by the lack of a standardized methodology at each step of the process and highlight the need for guidelines. Our review provides evidence that sicca patients seem to be more relevant than healthy subjects as a control group.

## Introduction

Sjögren’s syndrome (SS) is a systemic autoimmune disease typically affecting middle-aged women and is characterized by lymphocytic infiltration of the exocrine glands, resulting in severe exocrine sicca symptoms (1), such as dryness in the mouth (2). Oral features, such as early tooth loss, are predominantly caused by reduced salivary flow (3). Despite increasing knowledge on the pathogenesis of primary SS (pSS), its aetiology is still uncertain. Although many efforts have been made to find a systematic treatment, only symptomatic treatments are currently available (4).

Recently, microbiota has become a field of interest. Indeed, the Human Microbiome Project from the National Institute of Health in the United States characterized the microorganism compositions in several anatomical sites (5). The human microbiota is composed of 100 trillion bacteria, protozoa, fungi, and viruses. Microorganisms have established a symbiosis in different anatomical sites. However, dysbiosis, defined as an imbalance in microbiota, with an increase, a decrease or a change in the relative abundance of microorganisms (6), is suspected to be involved in the development of autoimmune diseases (7). Indeed, bacteria can cause autoimmune disease through different mechanisms, such as pathogen persistence, epitope spreading, molecular mimicry, epigenetic changes, and Toll-Like Receptor activation. For example, it has been demonstrated that the interaction of host cells in the intestine with commensal bacteria plays an important role in the development of Th17 and Treg lymphocytes (8), and may lead to auto-immune disease. Thus, many autoimmune diseases may be related to dysbiosis. For example, studies have demonstrated a link between the development of rheumatoid arthritis and the presence of *Porphyromonas gingivalis* (9) in the oral cavity many years before symptom onset, and this microorganism may be a predictive biomarker of the disease.

The gut and oral microbiota are the most abundant anatomical sites in terms of biodiversity, and they can harbor more than 1000 species (5). Currently, different techniques are used to analyze the oral microbiota: using oral saliva (based on oral washing, stimulated or unstimulated whole saliva) or buccal swabs of different parts of the oral cavity.

Currently, high-throughput methods for microbiome analysis, such as analysis of 16S rRNA or of the whole metagenome based on targeted or shotgun next-generation sequencing (NGS) techniques, have been developed (10) to sequence multiple DNA molecules at the same time. The diversity of a bacterial population is assessed by alpha- and beta-diversity. Alpha diversity corresponds to the within-individual diversity (measured by the Shannon or Simpson index) and the species richness (number of operational taxonomic units) of a bacterial population in one sample site. Beta-diversity (measured by the Bray–Curtis index, Jaccard distance, or UniFrac) and phylogenetic indexes (measured by the Faith index, for example) assess differences between samples; the latter does so by comparing taxonomic abundance profiles and incorporating phylogenetic differences between taxa. There are seven main taxonomic ranks: kingdom, phylum, class, order, family, genus, and species, from least precise to most precise. Taxonomic information about genera is more precise than that about phyla, for example.

Thanks to the development of these methods, studies have tried to investigate the link between microbiota and Sjögren’s disease (7). Previous animal’s studies had interesting results. For example, in a mice model of Sjögren syndrome (11), it has been demonstrated that the severity of SS ocular and systemic disease was inversely correlated with microbial diversity.

It has been hypothesized that gut and oral dysbiosis may contribute to the development of pSS. However, it is unclear whether dysbiosis is a consequence of sicca symptoms, such as a reduced salivary flow rate, or takes part in the pathophysiology.

Our main objective was to assemble the available data regarding the oral and gut microbiota in pSS and to compare them to those from healthy individuals, sicca patients or lupus disease patients to analyze frequent differences in the microbiota.

## Methods

### Information Sources and Searches

Systematic research in accordance with the PRISMA guidelines (12) was performed using the PubMed database and Google Scholar. All articles published from January 1, 2000, to June 1, 2020, were considered for inclusion. Literature published before this interval was excluded due to a lack of molecular techniques. The search queries were the following: [“Oral” OR “gut” OR “intestinal” AND “microbiota” OR “microbiome” OR “dysbiosis” OR “flora” OR “bacteria”] AND [“Sjögren” OR “pSS” OR “sicca”].

### Eligibility Criteria

The outcome was the evaluation of dysbiosis in pSS in terms of bacterial diversity (alpha and beta-diversity) and the comparison of the abundance of a particular taxon. Studies were included regardless of the oral or fecal sample techniques (rinsing samples, buccal swabs, etc.) or analysis techniques (bacterial culture, metagenomics, etc.).

Sjögren’s patients within the studies had to fulfil one of the classification criteria among the ACR/EULAR 2016 criteria (American College of Rheumatology/European League Against Rheumatism), the AECG (American-European Consensus Group) 2002 criteria and the ACR 2012 criteria. Studies with only secondary Sjögren’s patients were excluded. Studies with fewer than ten patients in total were excluded. Yeast-specific studies were excluded.

Reviews and *in vitro* studies were not included. Only studies written in English were included.

### Study Selection

Articles were selected by two independent reviewers (ED, GCA) considering their titles and abstracts according to the inclusion/exclusion criteria. Then, the selected articles were subjected to another analysis of their full text, and eligible articles were identified.

### Data Collection

The characteristics of the included studies were extracted using a standardized extraction. The extracted data were as follows: study identification, population, confounding variables, techniques of sampling, buccal information, methods for sample analysis, alpha- and beta-diversity, and differences in terms of phyla, genera, and species.

## Results

### Literature Search

**Figure 1:**
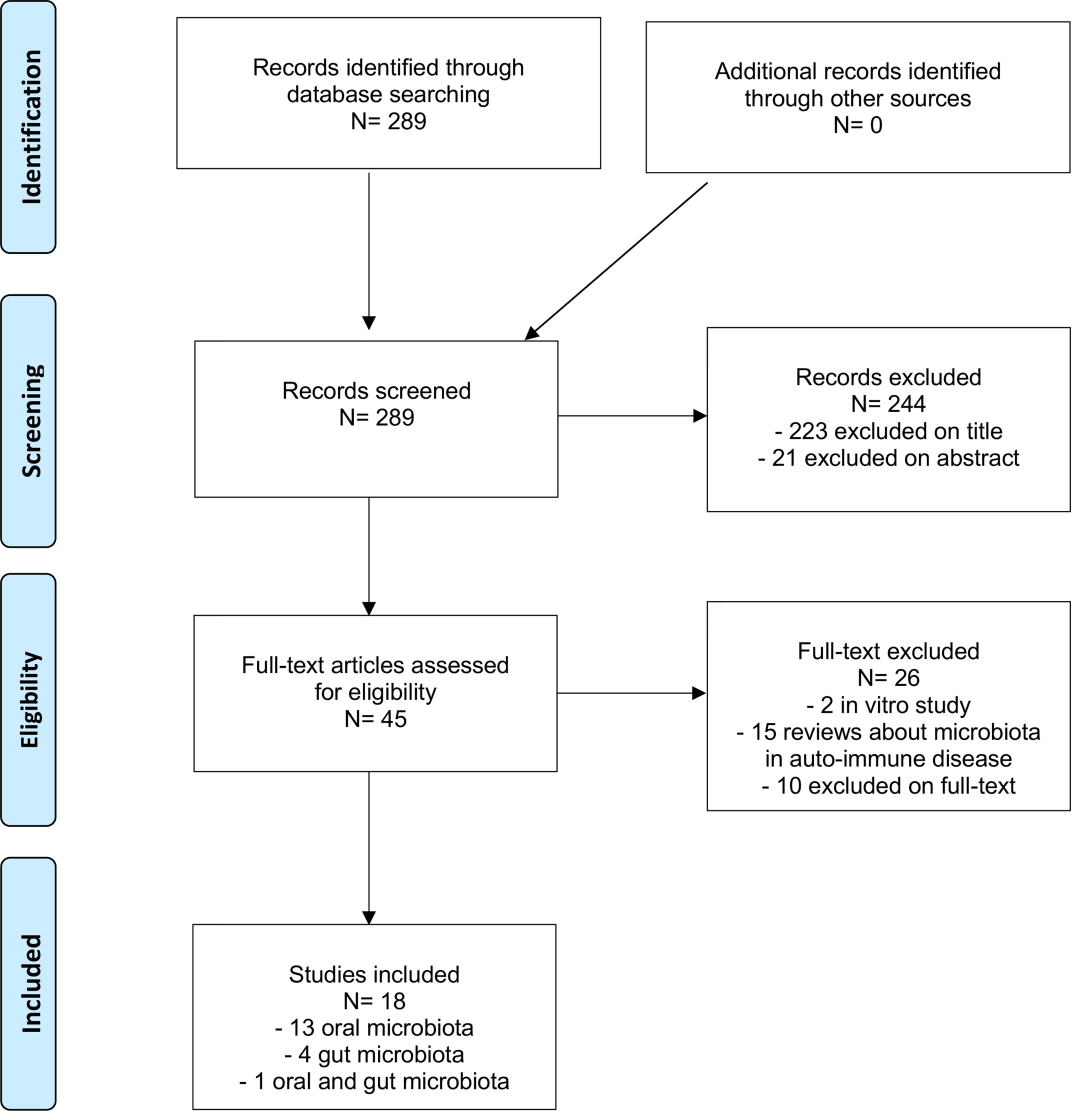
Flow-chart diagram of the selection process.

Overall, we identified 289 studies corresponding to our research aim. Finally, 18 studies were included after reading the full texts (**Figure 1**).

### General Patients’ Characteristics

Among the 18 studies included, 13 referred to the oral microbiota, 4 to the gut microbiota, and 1 to both anatomical sites. Their main characteristics of these studies are summarized in **Tables 1**, **2** and in **Supplementary Material 1**.

**Table 1:** Analysis of the methodology of the included studies relying on saliva samples in pSS patients compared to HCs and sicca patients.

Authors Year	Population	Confounding variables	Sampling		Analysis
			Liquid	Buccal hygiene	
Almstahl et al. (13)	14 Rx therapy 26 pSS 29 sicca 10 neuroL 36 HC	Different gender, smoking, treatment (Antibiotics, antifungal)	Oral washing	Yes (nb of teeth, plaque, periodontal probing pocket depth measure)	Selective culture media technique: Group Mutans Streptococci, *Lactobacillus*, *F. nucleatum, Prevotella* intermedia/*Prevotella* nigrescens, *Candida albicans*, *S*. *aureus*, Enterics
Leung et al. (14)	53 SS (26 pSS, 27 sSS) 29 HC	Similar age and gender Different treatment	SWS, SPS and SGP	Oral hygiene condition and periodontal status were similar	Selective culture media: - AGNR, Mutans streptococci, *Lactobacillus* and cultivable anaerobic flora of 11 SGP - Isolates that could not be identified were characterized using the MicroSeq 500 16S ribosomal DNA-based identification system.
Siddiqui et al. (15)	9 pSS 9 HC		UWS		The 16S rRNA hypervariable region V1-V2 was sequenced, which resulted in 106 614 raw reads.
van der Meulen et al. (16)	36 pSS 85 sicca 14 HC	Different age, gender, smoking, treatment	Oral washing	Nb of teeth	The 16S rRNA hypervariable region V4 was sequenced
Zhou et al. (17)	9 pSS 5 HC	Similar gender, age No antibiotics	UWS	Dental disease excluded	The 16S rRNA hypervariable region V4-V5 was sequenced
Zhou et al. (18)	22 pSS 23 HC	Similar gender and age	Oral washing	DMFT, DMFS, caries	The 16S rRNA hypervariable region V3-V4 was sequenced, which resulted in 2 579 826 raw reads
Rusthen et al. (19)	15 pSS 15 sicca 15 HC	Similar gender, age, smoking and dental status Different treatment	UWS, SWS	Yes (nb of teeth, DFM)	The 16S rRNA hypervariable region V3-V5 was sequenced, which resulted in 76 110 raw reads
Sembler-Moller et al. (20)	24 pSS 34 sicca	Similar age, gender, smoking status, treatment	UWS, SWS	Yes (DMFT, DMFS, dental plaque, gingivitis, or periodontal pocket depth)	The 16S rRNA hypervariable region V1-V3 was sequenced, which resulted in 1 404 081 sequences
Sharma et al. (21)	37 pSS 35 HC	No antibiotics, no smoking Similar gender	UWS	No extensive caries, use of dentures, oral ulceration, oral Candidiasis, or dental procedure in last 3 months	The 16S rRNA hypervariable region V3-V4 was sequenced
Alam et al. (22)	8 pSS without oral dryness 17 pSS with dryness 17 sicca 14 HC	No smoking, no antibiotics, steroids Similar gender, age	Oral washing		The 16S rRNA hypervariable region V1-V3 was sequenced

Rx, radiotherapy; pSS, primary Sjögren’s syndrome; HC, healthy controls; sicca, patients with dryness symptoms; neuroL, neuroleptics; nb, number; sSS, secondary Sjögren’s syndrome; SPS, parotid saliva; SGP, supra-gingival plaque; UWS, unstimulated whole saliva; SWS, stimulated whole saliva; DFMT, Decayed, Missing, and Filled Teeth index; DMFS, decayed, missing, and filled surfaces index; rRNA, ribosomal ribonucleic acid; AGNR, anaerobic gram-negative rods.

**Table 2:** Analysis of the methodology of the included studies relying on buccal swabs in pSS patients compared to HCs and sicca patients.

Authors Year	Population	Confounding variables	Sampling		Analysis
			Liquid	Buccal hygiene	
Almstahl et al. (23)	20 pSS 20 HC	Similar age, gender, nb of teeth Different smoking status, medication	Sterile cotton swabs on mucosa, dorsum of the tongue, supragingival tooth surfaces, gingival crevice region	Yes (UWS, SWS, nb of teeth, aspect of mucosa)	Selective culture media technique: Streptococci, S. *salivarus*, F. *nucleatum*, *Prevotella* intermedia/*Prevotella* nigrescens, *Candida albicans*, *S*. *aureus*, Enterics, Enterococci
Li et al. (24)	10 pSS : - 6 taking HY - 4 taking HY + PA 10 HC	Similar age, gender, no smoking, no antibiotics	Sterile cotton swabs on bilateral buccal mucosa	Yes (nb of teeth, aspect of mucosa, UWS, SWS)	The 16S rRNA hypervariable region V1-V3 was sequenced, which resulted in 366 452 raw reads
van der Meulen et al. (16)	37 pSS 86 sicca 24 HC matched with pSS patients on sex 103 patients from the general population	Similar age, smoking status Different treatment, gender	Sterile cotton swabs	Yes (nb of teeth, oral dryness, UWS, SWS)	The 16S rRNA hypervariable region V4 was sequenced.
van der Meulen et al. (25)	34 pSS 34 SLE	No antibiotics Different smoking status, age, gender	Oral washing and buccal swab collected at home by patients and frozen	WHO oral Health Questionnaire, Xerostomia inventory questionnaire	The 16S rRNA hypervariable region V4 was sequenced.

pSS, primary Sjögren’s syndrome; HC, healthy controls; sicca, patients with dryness symptoms; SLE, systemic lupus erythaematosus; UWS, unstimulated whole saliva; SWS, stimulated whole saliva; HY, hydroxychloroquine; PA, prednisone acetas; rRNA, ribosomal ribonucleic acid.

Each study included pSS according to validated classification criteria, with or without dry symptoms. The control population included healthy volunteers (13–19, 21–24, 26, 27), secondary Sjögren’s syndrome (sSS) patients (14) or patients with dry symptoms without a diagnosis of Sjögren’s syndrome (sicca) (13, 16, 19, 20, 22, 26). One study included controls with dry symptoms of different origins (i.e., hyposalivation caused by radiotherapy or neuroleptic treatment) (13). One study included patients with systemic lupus erythaematosus (SLE) (25).

Confounding variables, such as sex, age, and recent antibiotic intake, were considered in most studies. However, smoking status, medication intake and dental status were not always considered.

Oral samples consisted of unstimulated whole saliva in five studies (15, 17, 19–21). Two studies analyzed stimulated saliva (19, 20). Oral washing was performed in five studies (13, 16, 18, 22, 25). One study focused on parotid saliva and supra-gingival plaques (14), four studies focused on buccal swabs (23–26) and five studies focused on stool samples (25, 27–30). A buccal examination was carried out in seven studies (13, 14, 16, 18–20, 23). The Rome III diagnostic questionnaire on irritable bowel syndrome was completed in two studies centered on the gut microbiota (25, 28).

In total, 330 pSS patients with information on the oral microbiota and 120 pSS patients with information on the fecal microbiota were included.

### General Analysis Characteristics

The most common analysis method was 16S-targeted metagenomics, which was used in fourteen studies. The targeted 16S rDNA (i.e., *rrs* gene) region differed from one study to another. Three studies (13, 14, 23) used a culture-based approach with selective culture media that targeted different bacterial species. The storage, DNA extraction and PCR amplification techniques were highly heterogeneous between studies.

### Dysbiosis Features

The main results are summarized in **Tables 3**, **4** and in **Supplementary Material 2**.

**Table 3:** Analysis of the results of the included studies relying on saliva samples in pSS compared to HC and sicca.

Authors	Aim	Alpha diversity	Beta diversity	Phylum	Genus	Species in pSS
Almstahl et al. (13)	Analyse and compare the oral microflora in 4 groups of individuals with the same age, similar numbers of teeth and hyposalivation of different origins	Similar total microbial counts for pSS and HC				High number of *mutans Streptococcus*, *Candida albicans* and *Lactobacillus spp* in pSS
Leung et al. (14)	Microbiota of noncaries associated supra-gingival plaque microbiology in Sjögren’s syndrome in China compared to HC.					Higher *Lactobacillus* species in SWS and SGP culture. Lower proportion of gram-negative species in SGP
Siddiqui et al. (15)	Bacterial profile in whole saliva of pSS patients with a normal salivary flow rate by HTS compared to HC.	Lower species richness, alpha diversity in pSS compared to HC		Higher Firmicutes in pSS compared to HC Synergistetes and Spirochaetes were lower	*Streptococcus* was higher in pSS than in HC. 8 other taxa were lower in pSS than HC	Increase of *Veillonella* sp.*_*Oral_Taxon_917 Decreases of *P. gingivalis*, *Tannerella forsythia*, and *Treponema* sp._Oral _Taxon_237
van der Meulen et al. (16)	Oral microbiome pSS patients compared with sicca and HC.	No differences among the 3 groups	Large variation in bacterial composition in pSS and non-SS compared with HC		Lower *Streptococcus* and higher *Selenomonas* in pSS compared to HCs	
Zhou et al. (17)	Composition of the oral microbial flora in pSS patients and HC using HTS in China to provide guidance for treatment.	No difference between pSS patients and HC		16 phyla in total. Bacteroidetes and Firmicutes were more abundant in pSS. Proteobacteria was less abundant. No differences for Actinobacillus and Fusobacterium.	10 genera, including *Prevotella, Bacteroides* and *Actinomyces*, were more abundant in pSS patients. 17 genera, including *Neisseria*, were less abundant in pSS patients.	
Zhou et al. (18)	Oral microflora profile of pSS patients in the oral cavities by using HTS.	Lower oral bacterial community evenness and diversity in pSS patients compared to HC	No difference between pSS and HC		No difference between *Streptococcus* and *Lactobacillus.* *Veillonella* was higher in pSS than HC.	
Rusthen et al. (19)	Compare the salivary bacterial composition in pSS patients with sicca and HC to investigate a possible dysbiosis in pSS.	No differences among the 3 groups		No differences between groups.	No differences between groups for the most predominant genera. Only *Haemophilus* and *Neisseria* were decreased in pSS and sicca patients compared to HC.	*Porphyromonas pasteri* was decreased in pSS and sicca patients compared to HC.
Sembler-Moller et al. (20)	Characterize and compare the salivary microbiota in pSS and sicca and to relate the findings to their oral health status and saliva flow rates.	No differences between pSS and sicca				
Sharma et al. (21)	Evaluate the salivary microbiome in pSS patients using 16S rRNA sequencing approach.	No differences between pSS patients and HC. Low alpha diversity in patient with steroids			*Bifidobacterium*, *Dialister* and *Lactobacillus* were enriched, while *Leptotrichia* was depleted in pSS patients compared to HC	
Alam et al. (22)	Characterize the oral microbiota in SS patients and to investigate its potential role in the pathogenesis of SS.	Higher diversity in pSS patients compared to the control group with Shannon index, particularly in a non-dry condition		Increased Firmicutes and decreased Proteobacteria, Fusobacteria, TM7, and Spirochaetes in pSS patients	*Streptococcus*, *Prevotella*, *Lactobacillus*, *Atopobium*, and *Staphylococcus* were increased but 34 genera were underrepresented in SS patients compared to HC	Lower *Porphyromonas gingivalis* in pSS patients

pSS, primary Sjögren’s syndrome; HC, healthy controls; sicca, patients with dryness symptoms; SGP, supra-gingival plaques; SWS, stimulated whole saliva; HTS, high-throughput sequencing; rRNA, ribosomal ribonucleic acid.

**Table 4:** Analysis of the results of the included studies relying on buccal swabs in pSS patients compared to HCs and sicca patients.

Authors	Aim	Alpha diversity	Beta diversity	Phylum	Genus	Species
Almstahl et al. (23)	Composition of microbial flora in different sites in pSS and HC	Higher microbial density on the tongue Total microbial count on the dorsum of the tongue was similar in pSS and HC				Streptococci and S. *salivarius* were higher in pSS F. *nucleatum* was lower in pSS
Li et al. (24)	Investigate the oral microbiota in pSS patients as opposed to HC.			10 phyla were found. Proteobacteria were lower in pSS patients than HC.	339 genera were identified (248 for pSS patients, 270 for HC). *Ralstonia* prevalence was increased in pSS patients.	
van der Meulen et al. (16)	Assessed whether the microbiome of the buccal mucosa is specific for pSS patients compared with symptom-controls.	No differences between 3 groups	Higher variation in bacterial community in pSS patients compared to HC and sicca patients	Proteobacteria was lower in pSS patients compared with HC. Bacterial composition of pSS and sicca patients were comparable and differed from HC: a higher Firmicutes*/*Proteobacteria ratio	Lower *Streptococcus* and higher *Gemella* in pSS patients compared with sicca patients	
van der Meulen et al. (25)	Identify disease-specific differences in the gut and oral microbiota of pSS and SLE patients and assess whether pSS and SLE patients share overlapping signatures in gut microbiota composition.	Richness and diversity were higher in SLE patients compared pSS patients. The bacterial composition differed between pSS and SLE patients			No difference in *Capnocytophaga* in anti-Ro/SSA-positive patients (pSS and SLE patients together) compared with anti-Ro/SSA-negative patients	

pSS, primary Sjögren’s syndrome; HC, healthy controls; sicca, patients with dryness symptoms; SLE, systemic lupus erythaematosus.

#### pSS and Healthy Controls

##### Oral Microbiota

Ten studies focused on alpha-diversity with 236 pSS patients compared to 195 healthy controls in total. Among them, eight studies (13, 15–19, 21, 22) relied on saliva samples, and two studies (23, 26) relied on buccal swabs. Two studies found a lower species richness and alpha-diversity in pSS (15, 18) when using saliva samples. Other studies found no significant differences.

Three studies assessed beta-diversity with 95 pSS compared to 61 healthy controls in total. Among them, one study focused on oral washes and did not find significant differences (18). Two other studies, one using buccal swabs and the other using saliva samples, found a higher beta diversity in pSS (16, 26).

##### Gut Microbiota

Five studies analyzed alpha-diversity with 120 pSS compared to 1039 healthy controls in total. Among them, three showed decreased alpha-diversity in pSS (25, 27, 28). One study found no significant difference (29), while another study found no difference in Shannon’s diversity index, but Faith’s phylogenetic diversity showed an increased diversity in pSS (30).

Beta-diversity was analyzed in two studies and showed an increased beta-diversity in pSS (29) than in healthy patients in one study, an a decreased beta-diversity in another study (27).

#### pSS and Sicca Patients

Sicca mouth conditions could induce a modification of the local microbiota, so we analyzed the results comparing pSS patients and controls with sicca symptoms.

##### Oral Microbiota

Five studies assessed alpha-diversity with 137 pSS compared to 237 sicca patients in total. Four of them did not find significant differences in the microbiota between the groups (16, 19, 20, 26); one relied on buccal swabs (26), and the other relied on saliva samples (16, 19, 20), while one of them showed differences in the microbiota (22). This study used oral washing samples and showed an increased alpha diversity in pSS.

One study found a higher richness and diversity in buccal swabs and oral washings in SLE compared to pSS (25).

##### Gut Microbiota

Two studies assessed alpha-diversity with 23 pSS compared to 22 sicca patients in total. No significant difference was found in one study (29), while one study found an increased alpha-diversity in pSS compared to patients with dry eye symptoms (30).

One study analyzed beta-diversity and found a significant difference in pSS compared to patients with dry eye symptoms (29).

### Phyla, Genera and Species

#### pSS and Healthy Controls

##### Oral Microbiota

Five studies included information about microbiota at the phylum level. Two that relied on saliva samples found an increased relative abundance of Firmicutes (15, 17) among pSS. One study found a higher abundance of Bacteroidetes (17). Three studies found a decrease in Proteobacteria in pSS (17, 24, 26). Among these studies, two used buccal swabs samples. A higher Firmicutes/Proteobacteria ratio was found in one study (26).

Seven studies analyzed the microbiota at the genus taxonomic level. The results regarding *Streptococcus* were discordant. Indeed, one study found a higher relative abundance of *Streptococcus* in pSS (15), one found a lower relative abundance (16) and another found a similar abundance to that of a healthy population (18). Both studies used saliva samples for analyses. *Lactobacillus* had a higher abundance in one study (21) and showed no differences in another study (18). *Neisseria* was found to be decreased in two studies (17, 19) using unstimulated saliva samples. *Prevotella* and *Actinomyces* had a higher relative abundance in one study (17). *Veillonella* had increased abundance in one study (18).

Six studies included information at the species level. Among them, two studies found a higher level of *Lactobacillus* species in saliva samples (13, 14).

##### Gut Microbiota

Four studies assessed the phyla distribution. Three found a higher abundance of Bacteroidetes in pSS (25, 29, 30) compared to healthy controls, another one found a lower abundance (27). Firmicutes was decreased in two studies (29, 30), and the Firmicutes/Bacteroidetes ratio was lower in two studies (25, 29). However, one study did not find a significant difference in the Firmicutes/Bacteroidetes ratio when taking into account pSS and dry eye patients compared to healthy controls (30). Proteobacteria was increased in three studies (25, 27, 30). The results regarding Actinobacteria were discordant: two studies found a higher abundance (27, 30), while another study found a decrease in pSS (29).

Four studies analyzed microbiota at the genus level. One study found no difference between pSS patients and healthy controls in terms of genera (25). Another study showed that *Bifidobacterium*, *Blautia*, *Dorea* and *Agathobacter* were decreased in pSS patients (29). *Megasphaera*, *Parabacteroides* and *Prevotella* were found to be increased in pSS in one study (30). Another one (27) found a higher abundance of *Bacteroides*, *Prevotella* and *Bifidocaterium*.

Two studies included information at the species level. One study showed a lower level of species belonging to the *Bifidobacterium* and *Alistipes* genera in pSS (28).

#### pSS and Sicca Patients

##### Oral Microbiota

Three studies assessed microbiota at the phylum level. There were no differences among the nine major phyla in the two studies (19, 26) using saliva samples and buccal swabs. One study found a higher *Firmicutes* abundance in pSS and a decrease abundances of *Proteobacteria*, *Fusobacteria* and *Spirochaetes* (22).

Two studies presented results regarding genera. *Lactobacillus* were discordant in both studies. Indeed, one study found an increase in *Lactobacillus* in pSS (22) in saliva samples, while the other study found a decrease in buccal swabs (26). *Prevotella*, *Lactobacillus*, *Streptococcus* and *Staphylococcus* had higher relative abundance sin pSS in one study (22).

##### Gut Microbiota

One study found a higher level of *Bacteroidetes* and lower levels of *Firmicutes* and *Actinobacteria* in pSS (29).

## Discussion

Currently, only symptomatic treatments are available for pSS patients (4). However, pSS can have a strong impact on quality of life (31). Thus, the physiopathology of pSS is a major topic of interest to find new therapeutics. Microbiota has become an important field of interest and may lead to symptomatic treatments as well as causal treatments if its implications are confirmed.

In this systematic review, we identified a total of 330 pSS patients with information on their oral microbiota and 120 pSS patients with information on their fecal microbiota compared to a large number of controls. However, the results were mostly heterogeneous, and a clear tendency was not identified. In fact, the results regarding diversity were not always in accordance, and it is currently impossible to make certain conclusions about dysbiosis in pSS.

Moreover, the exploration of dysbiosis is complex, and many parameters must be considered. Indeed, the heterogeneous results might be explained by the lack of a standardized methodology at each step of the process, from sampling to the bioinformatics process.

Buccal swab techniques are heterogeneous in oral evaluations and include different regions. Each region of the oral cavity has a specific microbial flora. Zaura et al. (32) found that cheek samples were the least diverse area in terms of microbiota and that dental samples from approximal surfaces showed the highest microbial diversity in healthy individuals. In contrast, Lim et al. (33) found that the saliva collection method (spit, drool and oral rinse) did not influence the salivary microbiome profiles of healthy individuals.

For the gut microbiota, guidelines (34, 35) have been published to harmonize fecal evaluation but were not applied in the included studies and cannot be transposed to the oral microbiota.

In addition, the included studies mostly relied on the 16S-targeted metagenomics method because 16S is a universal and conserved gene. However, this method does not allow a full analysis of the genome compared to shotgun sequencing, which can explain the different results. In addition, 16S allows the analysis of the bacterial community only, and fungi cannot be analyzed. There are nine variable 16S regions (V1-V9), each suitable for primer binding. Clooney et al. (36) found that the factor responsible for the greatest variance in microbiota composition was the chosen 16S rDNA target methodology rather than the natural inter-individual variance. Thus, there is a risk of bias when comparing data generated using 16S rDNA targets. Population characteristics were not always considered, which can explain why some of the results of the included studies were discordant. Indeed, it is well known that sex (37) and age (38) can influence the microbiota. Sjögren’s disease mostly affects middle-aged women (1), and the majority of the included patients were female. Smoking status is also an important factor that influences microbiota. Wu et al. (39) showed that current smokers had a lower relative abundance of the phylum Proteobacteria than individuals who had never smoked. In this review, groups had similar smoking statuses in only six studies. Medication intake, particularly the intake of antibiotics, is also known to influence microbiota. Becker et al. (40) demonstrated that metronidazole and doxycycline could influence the bacterial richness and evenness of the gut microbiota in mice. Zhernakova et al. (41) found that the use of antibiotics was significantly associated with microbiome composition, in particular, with strong and significant decreases in two species from the genus *Bifidobacterium* (Actinobacteria phylum). However, only a few studies excluded patients with antibiotic intake. Immunosuppressants can also result in changes in the oral flora. Li et al. (24) investigated the effect of prednisone on the oral microbiota in pSS. They found that *Lactobacillus* and *Streptococcus* were more affected by corticosteroids than the disease itself. Van Der Meulen et al. (25) found that the use of NSAIDs explained 0.2% of the overall gut microbiota composition, suggesting that NSAIDs had a small effect on the gut microflora. Previous studies showed similar results (41, 42) with NSAIDS use.

Most studies evaluated the oral hygiene conditions through an oral examination and/or an oral questionnaire. Usually, pSS patients present with a higher prevalence of dental caries or periodontal problems (43). The stimulated secretion rate had a negative correlation with the number of aciduric microorganisms, i.e., bacteria grew well in acidic media, such as *Lactobacillus* or *Streptococcus* (44, 45), and salivary acidogenic microbial counts were considered an important indicator of dental caries risk. This could be an explanation for the higher prevalence of caries in pSS. Unexpectedly, most of the studies included in this systematic review did not find a higher number of caries in pSS, but patients made a regular dental care visit once the diagnosis was made (13, 20).

Interestingly, Siddiqui et al. (15) investigated the oral microbiota in pSS with normal salivation. They found a higher frequency of Firmicutes than healthy controls. Alpha-diversity was decreased in pSS. These findings could indicate a specific shift in microbiota independent of hyposalivation.

As previously emphasized, dysbiosis is not an answer and may be irrelevant to — and may even distract from — useful microbiome research (46). Profiling the microbiota of healthy and ill people is not sensible because a microbiota-based diagnosis is not needed to determine health status (46). One of the major strengths of this systematic review is that, in addition to healthy control populations, we focused on studies (n=8) that included patients with sicca symptoms, which is a validated control to assess the effect of Sjögren’s disease itself on microbiota. Indeed, patients with sicca symptoms presented oral and/or eye dryness without salivary gland involvement detected in histopathology or auto-antibodies (anti-SSA/Ro positive). It appeared that reduced salivary secretion explained more microbial changes than the underlying disease. Van Der Meulen et al. (16) showed that disease status explained 5% of the variation between samples, while stimulated whole salivary secretion explained 9% of the variation between samples, with significant differences. Sembler-Moller et al. (20) and Van Der Meulen et al. (25, 26) reached the same conclusion. It was demonstrated that hyposalivation was associated with the lack of clearance function and the buffering action of saliva as well as a decrease in antimicrobial salivary components (47). Dawes et al. (48) found that bicarbonate secretion was positively correlated with the salivary secretion rate. It is known that pH conditions can influence bacterial development. Takahashi et al. (49) revealed that *P. gingivalis* strains grew only at neutral pH, whereas strains of *Prevotella intermedia* could grow under acidic conditions. Thus, pH should be considered in further studies to find a relationship among hyposalivation, pH and microbial variation.

Szymula et al. (50) demonstrated in mice that Sjogren’s syndrome antigen A (SSA)/Ro60-reactive T cells are activated by peptides derived from the oral microbe *Capnocytophaga ochracea*, a gram-negative anaerobic bacterium usually present in gingival sites and dental plaques. These findings suggested a link between the microbiome and pSS by molecular mimicry theory. However, Siddiqui et al. (15) found no increased abundance of *C. ochracea* in salivary samples from patients with pSS with normal salivation, while it was affected in pSS in the Li et al. (24) study. In addition, Van Der Meulen et al. (25) showed no difference in the relative abundance of *Capnocytophaga* in anti-Ro/SSA-positive patients, including pSS and SLE patients together compared with anti-Ro/SSA-negative patients. Thus, further studies should be performed to confirm Szymula’s results.

De Paiva et al. hypothesized that reduced diversity may favour the emergence of pathogenic bacteria that disrupt the intestinal barrier and stimulate the production of inflammatory mediators (11). They found that the severity of SS ocular and systemic disease was inversely correlated with gut microbial diversity in mice. However, the results were discordant when comparing healthy individuals and pSS patients. Mendez et al. (30) found an increased diversity in pSS, and Moon et al. showed no differences in alpha diversity among groups, while Mandl et al. (28) and Van Der Meulen et al. (25) found a lower diversity in pSS. It is currently impossible to determine whether intestinal dysbiosis drives the inflammatory process or is only a consequence of systemic disease. However, it is known that T-helper 17 (Th17) and T-regulatory (Treg) cells are present in the intestinal mucosa, where they protect the host from pathogenic microorganisms and restrain excessive effector T-cell responses. Commensal bacteria can promote intestinal Treg cells, which can actively induce mucosal tolerance. As such, dysbiosis of the gut microbiome could be a consequence of imbalance of the Treg/Th17 axis and not a consequence of gut inflammation (51).

Few clinical trials for treating Sjögren’s syndrome with fecal microbial transfer (FMT), pre- or probiotics (monoclonal or polyclonal) have been done (52). For example, two studies (53, 54) showed that fecal microbiota transplant from conventional mice reverted dry eye phenotype in different mouse model of SS. Another study (55) evaluated probiotics capsule (Lactobacillus acidophilus, Lactobacillus bulgaricus, Streptococcus thermophilus and Bifidobacteriumbifidum) in pSS. There was a statistically significant reduction of the candidal load from baseline to the fifth week respectively, and no significant reduction in the placebo group. In addition, Wu et al. (27) evaluated the change in intestinal microbiota in pSS after therapy of Yangyin Yiqi Huoxue recipe (Radix Pseudostellariae 24 g, Radix paeoniae alba 18 g, Rhizoma Polygonati 18 g, Fructus Ligustri Lucidi 15 g, Rhizoma Dioscoreae 30 g, Fructus Schisandrae 10 g, Fructus Mume 15 g, and Rhizoma Rhodiolae sachalinesis 15 g, glabrous sarcandra herb 12 g). After the treatment, patients’ activity measured by ESSDAI score have decreased. These results are encouraging for future treatments.

In light of our work, it seems important to have a standardized methodology for future studies when evaluating the oral microbiota. For example, samplings should be homogenized, with a cotton swab of a unique area or with saliva sample (stimulated or unstimulated, with or without mouth rinsing). All patients should undergo an oral hygiene evaluation (number of teeth, salivary flow) because it can influence the results. Information regarding medication, smoking status, age, sex, and time to disease progression should be reported and adjusted to the results. A control population should be included. We found that a population of sicca patients seemed to be much more relevant as a control group than a group of healthy individuals, as it allowed us to avoid the mechanical effect of hyposalivation on the microbiota by focusing on the disease itself and not on its symptoms. NGS should be used with a standard 16S rRNA hypervariable region.

## Conclusion

Sjögren’s microbiota has been a topic of interest in recent years, and several studies have been published and confirmed that a dysregulated immune response against the normal microbiome is one of the pathways responsible for initiating autoimmune responses in Sjögren’s syndrome. Despite the large number of pSS patients and controls included in this systematic review, the results of the included studies are difficult to compare because of the lack of a standardized methodology. However, it seems that pSS patients have gut and oral dysbiosis with lower diversity. Currently, it is not possible to determine whether dysbiosis is a consequence of immune dysregulation or the principal origin of the disease.

Further studies with a more standardized methodology are needed to establish the role of the microbiome in the pathogenesis of pSS. Thus, prebiotics and probiotics could be a promising future therapy as a symptomatic treatment or a causal treatment.

## Data Availability Statement

The original contributions presented in the study are included in the article/**Supplementary Material**. Further inquiries can be directed to the corresponding author.

## Author Contributions

ED wrote the manuscript with support from GA, GH-A, and VD-P. ED and GA selected studies. VD-P supervised the project. All authors contributed to the article and approved the submitted version.

## Conflict of Interest

The authors declare that the research was conducted in the absence of any commercial or financial relationships that could be construed as a potential conflict of interest.

## Publisher’s Note

All claims expressed in this article are solely those of the authors and do not necessarily represent those of their affiliated organizations, or those of the publisher, the editors and the reviewers. Any product that may be evaluated in this article, or claim that may be made by its manufacturer, is not guaranteed or endorsed by the publisher.

## Supplementary Material

Supplementary Material 1 Analysis of the methodology of the included studies focusing on the gut microbiota in pSS patients compared to HCs and sicca patients. pSS, primary Sjögren’s syndrome; HC, healthy controls; sicca, patients with dryness symptoms; SLE, systemic lupus erythaematosus; IBS, irritable bowel syndrome; DHD, Dutch Healthy Diet index; FFQ, food frequency questionnaire; rRNA, ribosomal ribonucleic acid

Supplementary Material 2 Analysis of the results of the included studies focusing on the gut microbiota in pSS patients compared to HCs and sicca patients. pSS, primary Sjögren’s syndrome; HC, healthy controls; sicca, patients with dryness symptoms; SLE, systemic lupus erythaematosus
