# Supplementary material for: Healthy Patients Are Not the Best Controls for Microbiome-Based Clinical Studies: Example of Sjögren’s Syndrome in a Systematic Review
Source: Front Immunol. 2021 Jul 29;12:699011. doi: 10.3389/fimmu.2021.699011 (PMC8358393; doi:10.3389/fimmu.2021.699011)
Supplement: Supplementary Material 1 — Analysis of the methodology of the included studies focusing on the gut microbiota in pSS patients compared to HCs and sicca patients. pSS, primary Sjögren’s syndrome; HC, healthy controls; sicca, patients with dryness symptoms; SLE, systemic lupus erythaematosus; IBS, irritable bowel syndrome; DHD, Dutch Healthy Diet index; FFQ, food frequency questionnaire; rRNA, ribosomal ribonucleic acid [file DataSheet_1.docx]

**Supplementary material**

**Supplementary material 1:** Analysis of the methodology of the included studies focusing on the gut microbiota in pSS patients compared to HCs and sicca patients.

| **Authors** | **Population** | **Confounding variables** | **Sample** | | **Analysis** |
| --- | --- | --- | --- | --- | --- |
|  |  |  | **Material** | **Digestive** |  |
| T. Mandl et al.  2017 | 42 pSS patients  35 HC (age and sex-matched) | No antibiotics | Stool samples | Rome III diagnostic questionnaire on IBS, F-calprotectin | The GA-map™ Dysbiosis Test was used (use of 54 bacterial 16S rRNA probes specific).  Dysbiosis was defined as DIS ≥ 3, and severe dysbiosis as DIS 5. |
| TA Van Der Meulen et al.  2019 | 39 pSS patients  30 SLE patients  965 HC | Similar age, gender  No antibiotics, similar treatment  Different smoking status | Stool samples collected at home by patients and frozen | DHD, FFQ, Rome III diagnostic questionnaire on IBS | The 16S rRNA hypervariable region V4 was sequenced. |
| G. Wu et al. 2019 | 16 pSS  6 HC | Similar age, gender  Different smoking status, treatment | Fresh feces specimens |  | The 16S rRNA hypervariable region V3-V4 was sequenced. |
| J. Moon et al.  2020 | 10 pSS patients  14 dry eye patients  12 HC | Similar age, gender  No antibiotics  Different treatment | Stool samples collected at home |  | The 16S rRNA hypervariable region V3-V4 was sequenced. |
| R. Mendez et al.  2020 | 13 pSS patients  8 sicca patients  21 HC | Similar age, gender | Stool samples |  | The 16S rRNA hypervariable region V4-V5 was sequenced. |

pSS, primary Sjögren’s syndrome; HC, healthy controls; sicca, patients with dryness symptoms; SLE, systemic lupus erythaematosus; IBS, irritable bowel syndrome; DHD, Dutch Healthy Diet index; FFQ, food frequency questionnaire; rRNA, ribosomal ribonucleic acid

**Supplementary material 2:** Analysis of the results of the included studies focusing on the gut microbiota in pSS patients compared to HCs and sicca patients.

| **Authors** | **Aim** | **Alpha diversity** | **Beta diversity** | **Phylum** | **Genus** | **Species** |
| --- | --- | --- | --- | --- | --- | --- |
| T. Mandl et al.  2017 | Explore intestinal microbial balance in pSS patients and relate these findings to the clinical features of the disease. | Lower in pSS patients than HC |  |  |  | pSS patients showed decreased levels of bacteria from the genera *Bifidobacterium* and *Alistipes* compared to HC |
| TA Van Der Meulen et al.  2019 | Identify disease-specific differences in the gut and oral microbiota of pSS and SLE patients and assess whether pSS and SLE patients share overlapping signatures in the gut microbiota composition. | Alpha-diversity was similar between pSS and SLE patients but differ compared to HC  Richness was lower in pSS and SLE patients compared with HC, but diversity was similar among the 3 groups. |  | Bacteroidetes and Proteobacteria were higher in pSS and SLE patients compared with HC.  Lower Firmicutes/ Bacteroidetes ratio from pSS compared to HC | 6 genera were higher and 12 were lower in pSS and SLE patients compared with HC | *Bacteroides vulgatus*, *Bacteroides uniformis* and *Bacteroides ovatus* were higher in pSS and SLE patients than in HC. |
| G. Wu et al. 2019 | Explore the change of intestinal microecology in pSS and correlation with disease activity  Discuss the therapy effect of Yangyin Yiqi Huoxue Recipe | Lower in pSS than HC before treatment.  Increased alpha-diversity after treatment in pSS. | Lower in pSS than HC before treatment. | Higher proportion of *Actinobacteria*, *Firmicutes*,  *Fusobacteria*, and *Proteobacteria* in pSS.  Lower Bacteroidetes in pSS. | Higher proportion of *Bifidobacterium, Bacteroides,*  *Escherichia-Shigella, Faecalibacterium, and Prevotella* in pSS  Lower *Clostridia* in pSS. |  |
| J. Moon et al.  2020 | Investigate gut dysbiosis in patients with pSS or dry eye syndrome compared to normal subjects and evaluate the association of dysbiosis with dry  eye severity. | No differences among groups | Higher in pSS patients compared to 2 groups | Increased Bacteroidetes and decreased Firmicutes (lower Firmicutes/Bacteroidetes ratio), Clostridia and Actinobacteria in pSS patients compared to HC and sicca patients | pSS patients had decreased *Bifidobacterium*, *Blautia*, *Dorea* and *Agathobacter*  compared to controls and increased *Prevotella*, *Odoribacter* and *Alistipes* compared to dry patients |  |
| R. Mendez et al.  2020 | Evaluate the diversity, dimensionality and constituency of the gut microbiome in individuals with dry eye population and to correlate gut  microbiome profiles to clinical parameters of disease. | Shannon’s diversity index showed no differences among groups  Faith’s phylogenetic diversity showed increased diversity in pSS patients compared to controls |  | No difference between  pSS+sicca patients and HC in terms of the Bacteroides/Firmicutes ratio.  Less Firmicutes and higher Proteobacteria, Actinobacteria and Bacteroidetes in pSS+sicca patients | Increase in *Megasphaera*,  *Parabacteroides* and *Prevotella* in pSS patients compared to HC |  |

pSS, primary Sjögren’s syndrome; HC, healthy controls; sicca, patients with dryness symptoms; SLE, systemic lupus erythaematosus
